# Supplementary material for: Programmable CRISPR interference for gene silencing using Cas13a in mosquitoes
Source: J Genomics. 2020 Mar 1;8:30–6. doi: 10.7150/jgen.43928 (PMC7064736; doi:10.7150/jgen.43928)
Supplement: Supplementary file 1 — Supplementary figures and tables. [file jgenv08p0030s1.pdf]

Table S1. Primer sets used in the study

Primer	Sequence	Amplicon Size (bp)
Aa_Cactus_F	AGACAGCCGCACCTTCGATTCC	232
Aa_Cactus_R	CGCTTCGGTAGCCTCGTGGATC	
Aa_Caspar_F	GAATCCGAGCGAGCCGATGC	270
Aa_Caspar_R	CGTAGTCCAGCGTTGTGAGGTC	
Aa_rpS7_F	AAGGTCGACACCTTCACGTC	252
Aa_rpS7_R	TCTTGTCCCTCCGTTTGGTG	
Aa_rpS17_F	CGTGAGCGCAGAGACAACTA	240
Aa_rpS17_R	ACCATGGATGTTCCGGTGTG	
Aa_COPI_F	GACGTTGCGCATATCAGACG	256
Aa_COPI_R	CAGCATTCTCAGAGGGCCAA	
Cas13a_F	TCCGCCAACAAGGAAGAGAC	442
Cas13a_R	CCGATGGCCTTCTCGTACTC	
Ag_Vg_F	ACTTCTTCCAGGGCAAGCAC	250
Ag_Vg_R	CACAGCGCAAGATGGATGGT	
Ag_ rpS7_F	GCGTGAGGTCGAGTTCAACA	211
Ag_ rpS7_R	GGGAACGCGGTCTCTTCTG	
Ag_ rpS5_F	CCATGTCACGTCTCGTCACT	104
Ag_ rpS5_R	CGAAAACCATCCACACACAC	

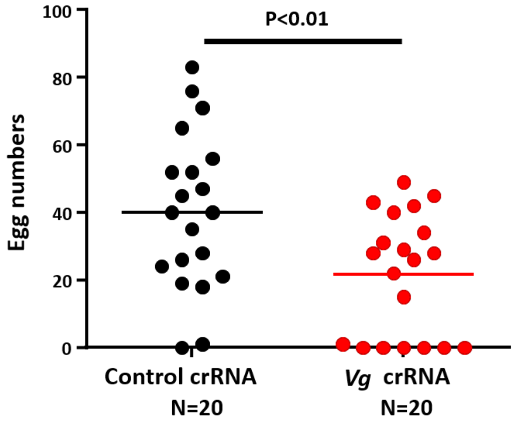

Figure S1. The Cas13a/*Vg* crRNA treatment significantly reduced egg production in *An. gambiae.* Mann-Whitney test, P<0.01.

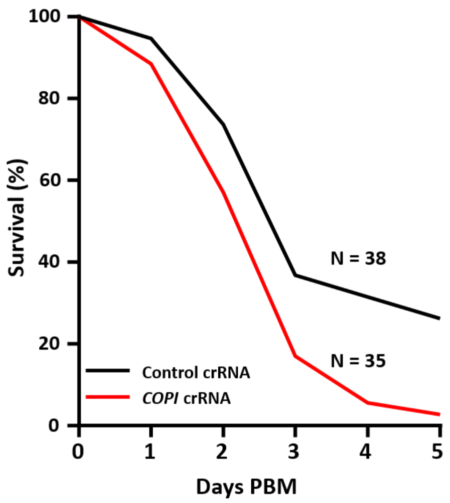

Figure S2. The Cas13a/*COPI*-crRNA treatment resulted in a significant higher mortality post a blood meal. Mantel Cox test, P<0.01.
